# Kaposi Sarcoma Presenting as Upper Gastrointestinal Bleeding in a Patient With Acquired Immune Deficiency Syndrome

**DOI:** 10.1155/crgm/7703200

**Published:** 2025-05-27

**Authors:** Karthik Gnanapandithan, Mohammad T. Hussain, Daniel Kashani, Philip N. Okafor

**Affiliations:** ^1^Division of Hospital Internal Medicine, Mayo Clinic, Jacksonville 32224, Florida, USA; ^2^Department of Gastroenterology, Mayo Clinic, Jacksonville 32224, Florida, USA

**Keywords:** acquired immune deficiency syndrome, human immunodeficiency virus, Kaposi sarcoma, upper gastrointestinal bleeding

## Abstract

Kaposi sarcoma (KS), an angioproliferative neoplasm driven by human herpesvirus 8, predominantly affects patients with acquired immune deficiency syndrome (AIDS) or those on immunosuppressive therapy. Gastrointestinal involvement in KS is underreported, with limited literature highlighting its clinical significance and morphological diversity on endoscopy. This case report illustrates the complexities of diagnosing and managing gastrointestinal KS in an AIDS patient who presented with upper gastrointestinal bleeding. The diagnosis was established through the characteristic endoscopic appearance of the lesions, supported by histopathological confirmation. This case emphasizes the variable endoscopic manifestations of KS, ranging from linear ulcers to nodular lesions, and underscores the necessity for heightened clinical vigilance and multiple deep biopsies to avoid false-negative results. Treatment options, primarily palliative, include highly active antiretroviral therapy, chemotherapy, and radiation, yet the prognosis remains poor with high short-term mortality. This report contributes to the sparse literature on gastrointestinal KS, advocating for increased awareness and early intervention to potentially improve outcomes in this patient population.

## 1. Introduction

Kaposi sarcoma (KS) is an angioproliferative disorder with multiorgan involvement linked to human herpesvirus 8 (HHV-8) infection. There are two commonly seen clinical variants of KS: one associated with acquired immune deficiency syndrome (AIDS) and the other is iatrogenic KS associated with immunosuppression. KS in patients with AIDS usually follows a more aggressive clinical course with widespread organ involvement, including the skin, mucus membranes, lymphatics, and visceral organs [1]. Patients with human immunodeficiency virus (HIV)–associated KS often require highly active antiretroviral therapy (HAART) along with systemic chemotherapy, and the prognosis is grim unless immune reconstitution is achieved. In contrast, immunosuppression-associated KS and some other non-HIV KS tend to have an indolent behavior [2]. In the latter cases, withdrawal of immunosuppression alone can lead to resolution of mucocutaneous KS, while those with involvement of other organ systems respond favorably to systemic chemotherapy [2, 3]. While cutaneous manifestations are well-documented, gastrointestinal (GI) involvement is less frequently recognized and presents unique diagnostic and therapeutic challenges. This case report details the presentation and management of a patient with AIDS who developed upper GI bleeding as a complication of KS, highlighting its endoscopic diversity, diagnostic hurdles, and the critical need for an integrated approach to care.

## 2. Case Report

**Figure 1:**
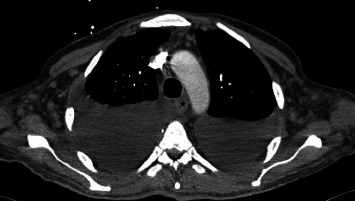
CT of the chest showing mediastinal and axillary lymphadenopathy with bilateral pleural effusions.

**Figure 2:**
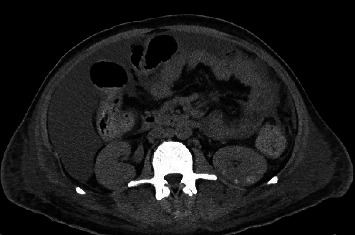
CT of the abdomen showing diffuse irregular and nodular gastric wall thickening.

**Figure 3:**
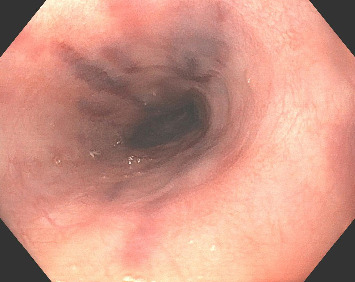
EGD with linear hemorrhagic mucosal erosions in the mid-esophagus.

**Figure 4:**
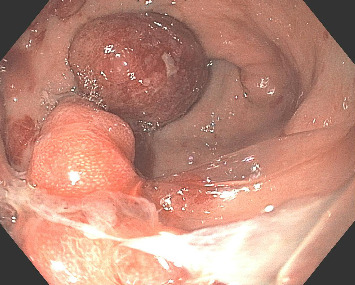
EGD reveals large polypoid hemorrhagic masses in the body of the stomach.

**Figure 5:**
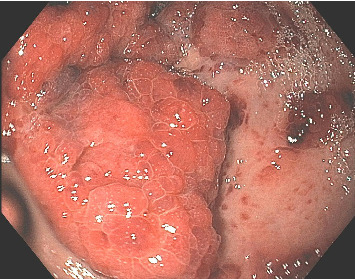
EGD showing friable mucosa and nodular masses in the gastric antrum with stigmata of recent bleeding.

A 37-year-old male with a history of HIV infection with AIDS presented to the emergency department with dysphagia, coffee-ground emesis, black stools, and fatigue. He was diagnosed with HIV about 4 years back and had no other medical history or relevant family history. He was managed in another facility for his AIDS and was poorly compliant with treatment and follow-up. He had been hospitalized a few times in the last year with several infections, including *Pneumocystis jirovecii* pneumonia and *Cryptosporidium gastroenteritis*. Given his declining functional status, he had chosen to pursue hospice care about 6 months prior and had stopped all medications. He reported prior episodes of upper GI bleeding and had undergone esophagoduodenoscopy (EGD) 3 months back in another facility, but the results were unavailable. Since then, he has had progressive dysphagia, anorexia, and intermittent black stools. His hemoglobin on presentation was 7.4 g/dL. HIV RNA load was 188,000 copies/mL, and CD4 count was undetectable. Computerized tomography (CT) of the chest (Figure 1) showed bilateral pleural effusions with axillary and mediastinal lymphadenopathy. CT abdomen (Figure 2) showed irregular gastric wall thickening and signs of blood products. He required blood transfusions for ongoing bleeding and was taken for EGD. EGD showed scattered mucosal erosions in the upper and middle third of the esophagus with a linear hemorrhagic appearance (Figure 3). There were multiple large polypoid hemorrhagic masses (Figures 4 and 5) with stigmata of recent bleeding in the stomach, and one of the lesions was partially obstructing the gastric outlet.

Morphological findings were highly suspicious of KS. Superficial biopsies taken from the esophagus and the gastric antrum revealed no evidence of KS with negative immunostaining for HSV and CMV. A review of his outside medical records showed that he was diagnosed with KS about 4 months back when he had presented with similar symptoms. Initial EGD had shown multiple nodular lesions in the stomach, and superficial biopsy at that time was negative for KS. A repeat endoscopy was performed a week later, with multiple deeper biopsies that showed lamina propria congestion and proliferation of vascular and spindle cells, along with extravasated blood cells, suggestive of KS. Immunohistochemistry confirmed the diagnosis, showing diffuse nuclear positivity for HHV-8 in the spindle cell proliferation. Bleeding had resolved, and he was recommended to continue HAART on discharge. However, he opted for discharge on comfort care and subsequently passed away within the next 2 weeks. The pathology slides from the other institution were unavailable for publication.

## 3. Discussion

In patients with HIV, the differential diagnosis of GI bleeding extends beyond common etiologies to include complications from opportunistic infections and malignancies. Apart from usual causes such as peptic ulcers and esophagitis, clinicians must consider ulcerations from cytomegalovirus or herpes simplex virus, non-Hodgkin's lymphoma, and AIDS-defining lesions such as KS or bacillary angiomatosis [1]. KS, often associated with AIDS, can also manifest in patients undergoing immunosuppressive treatments. It represents the most frequent GI malignancy in the AIDS population, surpassing lymphoma in prevalence. The GI tract is the most affected extracutaneous site, with involvement seen in up to 40% of KS patients [4]. In HIV patients, GI involvement is more common in those with low CD4 count and high viral load [5].

KS was first described in 1872 by a dermatologist, Moritz Kaposi, who called it “idiopathic multiple sarcomas of the skin [6].” GI involvement by KS may occur even in the absence of cutaneous manifestations, illustrating the disease's insidious nature. Clinically, the spectrum of symptoms is broad, ranging from abdominal pain, nausea, vomiting, and diarrhea to more severe manifestations like GI bleeding or intestinal obstruction. The endoscopic presentation of KS lesions in the GI tract is highly variable, displaying characteristics from linear ulcers and flat macular lesions to polypoid or nodular formations, often accompanied by submucosal hemorrhage [7, 8]. This is similar to the skin lesions in KS, which can also take on a widely varied pattern, including patches, nodules, or plaques [9]. Histopathological examination remains the gold standard for diagnosis. However, the submucosal origin of the tumor presents a diagnostic challenge, as superficial biopsies frequently yield false-negative results. A study highlighted a 35% false-negative rate in EGD biopsies for patients with GI KS [7]. The adoption of multiple deep biopsies has been recommended to improve diagnostic accuracy, and the risk of post-biopsy bleeding is considered minimal despite the vascular nature of KS lesions [4].

Therapeutically, endoscopic interventions employed for peptic ulcer disease, such as epinephrine injection or the application of hemostatic clips, can effectively manage vascular lesions with active bleeding. In most patients, the goal of KS treatment is palliation, focusing on symptom relief and reducing disease progression. The therapeutic arsenal includes HAART, radiation therapy, and chemotherapy, used singularly or in combination [7]. HAART serves as the cornerstone and the initial therapeutic approach. The treatment decision integrates factors such as the severity of HIV infection and the stage of KS. For patients with extensive disease involvement, systemic chemotherapy, including liposomal anthracyclines like doxorubicin, is considered [10]. Antiangiogenic therapy and immune checkpoint inhibitors have recently shown promising results in managing systemic KS, including those with GI involvement [11].

Despite therapeutic advances, the prognosis for AIDS patients with GI KS remains dire. The severity of the disease, compounded by the challenges in managing advanced HIV, contributes to a high mortality rate, with studies indicating a 6-month mortality exceeding 50% [12]. This underscores the necessity for early recognition and aggressive management of KS in HIV-infected individuals.

## 4. Conclusion

This case highlights the diagnostic challenges and clinical severity of GI KS in the setting of advanced AIDS. Early endoscopic evaluation with multiple deep biopsies is essential for diagnosis, and timely initiation of HAART remains the cornerstone of management. Multidisciplinary care and heightened clinical suspicion are critical to improving outcomes in this vulnerable population.

## Data Availability

The authors have nothing to report.
